# Comparison of mechanical resuscitation by an LV Impella device to extracorporeal resuscitation using VAECMO in a large animal model

**DOI:** 10.1038/s41598-025-93264-2

**Published:** 2025-03-19

**Authors:** Sebastian Billig, Adomas Kanauskas, Alexander Theißen, Nadine Hochhausen, Siarhei Yelenski, Katharina Nubbemeyer, Christoph Nix, Eveline Bennek-Schoepping, Matthias Derwall

**Affiliations:** 1 Department of Anesthesiology, RWTH Aachen University, Aachen, Germany; 2 Department of Thoracic Surgery, RWTH Aachen University, Aachen, Germany; 3 Abiomed Europe GmbH, Aachen, Germany; 4 Department of Anesthesia, Critical Care and Pain Medicine, St. Johannes Hospital, Dortmund, Germany

**Keywords:** ECPR, ECMO, Impella, Cardiac arrest, Mechanical circulatory support, Resuscitation, Preclinical research, Cardiovascular diseases, Cardiac device therapy

## Abstract

Extracorporeal cardiopulmonary resuscitation (ECPR) is an effective treatment for cardiac arrest (CA). Percutaneous left ventricular (LV) assist devices such as the Impella ECP (intravascular CPR [ICPR]) have been proposed as a less invasive alternative. The aim of this study was to explore the haemodynamic differences between ECPR and ICPR using a large animal model of electrically induced CA. Fourteen juvenile female German landrace pigs (72.4 ± 9.8 kg) were subjected to electrically induced CA for 5 mins followed by either ECPR (veno-arterial extracorporeal membrane oxygenation [VA-ECMO]) or ICPR (Impella ECP). Haemodynamic parameters and echocardiographic ventricular function indicators were monitored. Mechanical circulatory support (MCS) was continued until five hours after the return of spontaneous circulation (ROSC), when the devices were removed. Resuscitation outcomes and the haemodynamic effects of ECPR and ICPR were compared. The cannulation time for ECMO (469 ± 129 s) was significantly longer than the time for Impella device implantation (153 ± 64 s, *p* < 0.001). ECPR facilitated ROSC in 6/6 animals, whereas ICPR facilitated ROSC in 6/8 animals (*p* = 0.19). Echocardiography revealed no difference in LV or right ventricular (RV) dysfunction between the ECPR- and ICPR-treated animals after resuscitation (LV-global longitudinal strain [GLS] 3 h post-ROSC: ICPR: − 16.5 ± 5.6% vs. ECPR: − 13.7 ± 5.9%, *p* = 0.99; RV-GLS 3 h post-ROSC: ICPR: − 15.9 ± 3.3% vs. ECPR: − 17.3 ± 10.6%, *p* = 0.99). MCS using VA-ECMO and the Impella device both provided effective haemodynamic support during CA and post-ROSC in this large animal model. Despite LV unloading conferring a hypothetical advantage for ICPR, no significant differences in myocardial recovery were observed.

## Introduction

Extracorporeal cardiopulmonary resuscitation (ECPR), i.e., mechanical circulatory support (MCS) using veno-arterial extracorporeal membrane oxygenation (VA-ECMO), during cardiac arrest (CA) has successfully been implemented in the clinical practice of cardiopulmonary resuscitation (CPR)^1,2^. Recent studies have indicated that ECPR in comparison to conventional chest compressions may result in superior survival rates and a more favourable neurologic outcome in selected patients^3,4^. In addition to the rise of ECPR, percutaneous left ventricular assist devices (pLVADs) have been proposed as an option for MCS during resuscitation (intravascular CPR [ICPR]) instead of chest compressions, with promising results^5–9^.

In principle, ICPR could offer several advantages over ECPR due to its distinct mode of operation: the direction of physiological blood flow generated by transaortic pLVADs leads to left ventricular (LV) unloading and antegrade blood flow in the ascending aorta. In contrast, peripheral cannulated ECMO generates retrograde, non-physiological blood flow in the aorta, increasing LV afterload and ventricular work^10^. After the return of spontaneous circulation (ROSC), the function of the myocardium is often severely impaired^11,12^. VA-ECMO can negatively affect LV function through ECMO-generated afterload, which may further restrict LV function and hinder recovery. Furthermore, ECPR requires at least two large-bore vascular cannulas, increasing the risk of severe complications such as limb ischaemia or major bleeding^13^. On the other hand, ICPR requires a single vascular access point with a smaller diameter, potentially reducing the vascular access time and complication rate. However, a noteworthy limitation of ICPR is its inability to generate an active right ventricular (RV) output. Consequently, systemic perfusion during ICPR depends on Fontan-like passive pulmonary circulation, which enables LV filling. Therefore, the aim of our study was to explore the differences in resuscitation and post-resuscitation haemodynamics between ECPR and ICPR, utilizing a well-established large animal model of electrically induced CA^5,14–16^.

## Methods

This trial was conducted in 14 juvenile female German landrace pigs. Eight swine were resuscitated using a pLVAD (expandable Impella CP [Impella ECP], Abiomed Europe GmbH, Aachen, Germany) (74.2 ± 6.7 kg), and six were resuscitated using VA-ECMO (70.0 ± 13.3 kg). The Impella ECP pLVAD is a novel, self-expandable microaxial pump. The procedural methodology for device implantation has been presented in a separate publication^17^. The experimental design received approval from the responsible governmental institution (approval no. 84-02.04.2017.A300; North Rhine-Westphalia Office of Nature, Environment and Consumer Protection, LANUV NRW). The experiments comply with the ARRIVE guidelines and were carried out in adherence to the EU Directive 2010/63/EU for animal experiments.

### Preparation

After sedation with intramuscular 6 mg/kg azaperone (Stresnil, Janssen-Cilag GmbH, Neuss, Germany), the animals underwent ear vein cannulation, and narcosis was induced (2 mg/kg propofol, propofol 1% MCT, Fresenius Kabi Austria GmbH, Graz, Austria; 5 µg/kg fentanyl, Fentanyl-Jannsen, Janssen-Cilag GmbH). Anaesthesia was maintained throughout the whole experiment by propofol (5 mg/kg/h) and fentanyl (5 µg/kg/h), accompanied by Ringer’s solution at an infusion rate of 4 mL/kg/h. Mechanical ventilation (Cato, Dräger, Lübeck, Germany) via an endotracheal tube (inspired oxygen fraction, 0.3; tidal volume, 10 mL/kg; positive end-expiratory pressure, 5 cmH_2_O) was implemented. We adapted the respiratory frequency to preserve physiologic end-tidal carbon dioxide levels (35–40 mmHg). Body temperature was maintained at 38.2 ± 0.2 °C using convective heating (Warm Touch 5300A, Covidien, Dublin, Ireland).

**Fig. 1:**
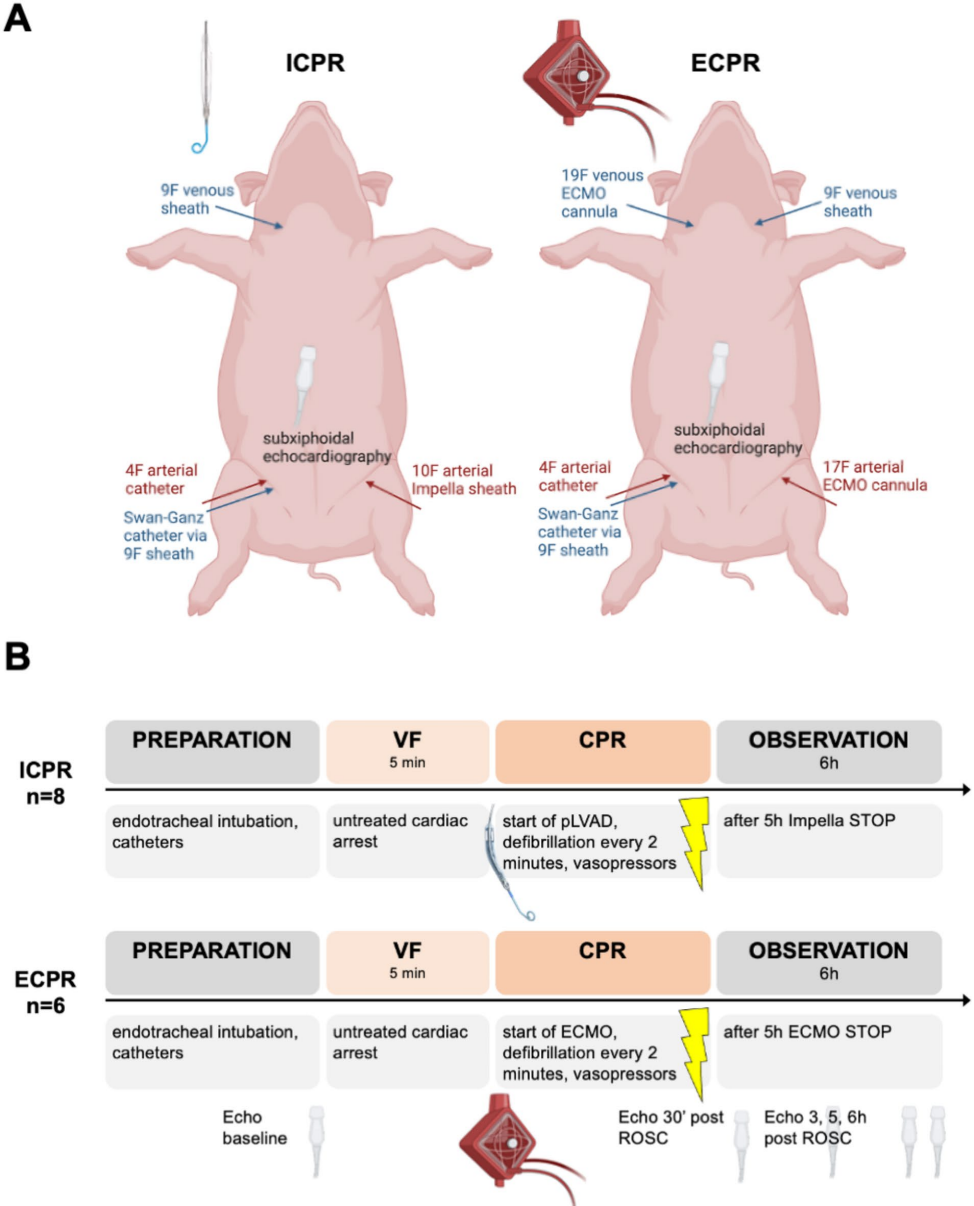
Illustration of the experimental setup. (**A**) Animals were prepared as illustrated here and received either resuscitation by an Impella pLVAD (ICPR) or veno-arterial ECMO (ECPR). (**B**) The flowchart visualizes the individual steps of the experiment: after completion of the preparation, the animals were exposed to electrically induced cardiac arrest for 5 min via the induction of ventricular fibrillation (VF). Depending on the treatment group, Impella (ICPR) or veno-arterial ECMO (ECPR) was then used for resuscitation. After the return of spontaneous circulation (ROSC), the animals were observed for 5 h until the devices were stopped, and the animals were sacrificed 6 h post-ROSC. Echocardiography was performed at the indicated time points during the experiment. *CPR* cardiopulmonary resuscitation; *ECMO* extracorporeal membrane oxygenation; *pLVAD* percutaneous left ventricular assist device; *ROSC* return of spontaneous circulation; *VF* ventricular fibrillation (created with *BioRender.com*).

A venous sheath (9F percutaneous sheath introducer set, Arrow, Reading, PA, USA) in the right femoral vein was used to advance a hexalumen Swan-Ganz catheter (744HF75, Edwards Lifesciences, Irvine, CA, USA) connected to a Vigilance VGS2 monitor (Edward Lifesciences) into the pulmonary artery. A 4F arterial catheter (arterial leadercath; Vygon, Ecquen, France) was inserted into the right femoral artery. Subsequently, 100 IU/kg heparin (B. Braun, Melsungen, Germany) was administered. Defibrillation electrodes (QUIK-COMBO, Physio-Control, Redmond, WA, USA) were attached to the animals and linked to a defibrillator (Lifepak 12, Medtronic, Minneapolis, MN, USA). Depending on the experimental group, the animals received either ICPR or ECPR for MCS during and after resuscitation (Fig. 1), as described below.

*ICPR* (Impella ECP): A 10F introducer sheath (10F Introducer Avanti; Cordis, Miami Lakes, FL, USA) was used in the left femoral artery, and a 9F sheath was used in the right jugular vein.

*ECPR* (VA-ECMO): A 19F ECMO cannula (Venous HLS Cannula PVS 1938, Maquet Cardiopulmonary GmbH, Rastatt, Germany) in the right jugular vein, a 17F ECMO cannula (Arterial HLS Cannula PAS 1715, Maquet) in the left femoral artery, and a 9F sheath in the left jugular vein were used. The ECMO cannulas were flushed and connected to the ECMO circuit (Maquet Cardiohelp, Intervention set BIOLINE COATING standard, Maquet). The oxygenator was connected to a heat exchanger set to 38.2 °C The ECMO cannulas remained clamped during preparation. We measured the cannulation time i.e. the time from the needle entering the skin for arterial or venous puncture until final sheath or cannula placement for both experimental groups.

### Experimental protocol

Following an equilibration period, the animals received a second injection of 100 IU/kg heparin. After electric induction of ventricular fibrillation (VF), ventilation and fluid administration were ceased. The CA remained untreated for 5 min. Resuscitation was then initiated by ventilation with 100% oxygen and the administration of Ringer’s solution at a rate of 200 mL/min. To ensure comparable no-flow times in the ICPR and ECPR groups, a non-randomized approach was employed, and ICPR experiments were performed first. After 5 min of untreated CA, the Impella ECP device was introduced via the previously placed femoral introducer sheath, and resuscitation was performed. The experiments in the ECPR group were subsequently conducted with the initiation of VA-ECMO after a total of 6 min of CA to achieve comparable no-flow times to those in the ICPR group. No animals in either group received chest compressions. MCS was initiated according to the respective treatment protocol for ICPR or ECPR:

*ICPR*: Immediately after fluoroscopic guided placement, the Impella device was activated with the maximum achievable flow. Norepinephrine (Arterenol; Sanofi, Frankfurt, Germany) (1 mg) was administered. An electric shock (360J, biphasic) was administered after 2 min of MCS and repeated every 2 mins if unsuccessful. If VF persisted after the second shock, another dose of norepinephrine was administered, and resuscitation was continued for another 2 min until the next shock. This protocol was continued until either 10 min of CPR had elapsed or ROSC was achieved. When ROSC was observed the ventilator settings from the preparation phase were resumed and fluid administration was limited to 4 mL/kg/h. No vasopressors were administered after ROSC. The animals were monitored for another five hours, when the Impella device was removed. Following observation for one hour after device removal, the animals were sacrificed by an intravenous administration of pentobarbital.

*ECPR*: ECMO treatment was initiated 60 s after the start of resuscitation to synchronize the ischaemia time with that in the ICPR group. Then, the cannula clamps were released, and VA-ECMO was initiated (F_i_O_2_ = 1.0; 1 L/min sweep gas flow; 3 L/min targeted blood flow). Vasopressor treatment and defibrillation were conducted using the same protocol as that used in the ICPR group. Following ROSC, the ECMO flow was reduced to a targeted blood flow of 2.5 L/min, the sweep gas flow F_i_O_2_ was reduced to 0.5, and the ventilator the ventilator settings from the preparation phase were resumed. No vasopressors were administered after ROSC. ECMO weaning and cannula clamping were conducted 5 h after ROSC. The animals were observed for another hour without ECMO support before the animals were euthanized by an intravenous administration of pentobarbital.

### Measurements

A small subxiphoidal access point was prepared for repeated echocardiographic examinations. Echocardiography was conducted using a transthoracic ultrasound probe (4Vc-D, GE Healthcare Chicago, IL, USA) attached to a GE Vivid E9 system (GE Healthcare). Echocardiography was carried out at the following time points: baseline; 30 min post-ROSC; and 3 h, 5 h and 6 h post-ROSC. Body surface area (BSA) indexed values were calculated using the following formula for BSA estimation: BSA = 0.0734 × bodyweight^0.656^^18^

Continuous monitoring via electrocardiogram (ECG), pulse oximetry and cerebral oximetry-near-infrared spectroscopy (NIRS) (INVOS 5100c cerebral oximeter; INVOS cerebral oximetry adult sensors, Medtronic, Dublin, Ireland) was conducted throughout the experiment. Femoral, pulmonary arterial and central venous pressures were monitored throughout the study (LabVIEW 2010, National Instruments, Austin, TX, USA; RedLab 1616HS-BNC, Meilhaus Electronic, Puchheim, Germany). The coronary perfusion pressure (CPP) was estimated by subtracting the mid-diastolic right atrial pressure from the mid-diastolic femoral pressure^19^. Owing to the lack of pulsatility in the blood pressure signal of the non-contracting ventricle during resuscitation, we used the mean right atrial pressure and the mean femoral pressure for this measurement. Arterial and mixed venous blood samples were drawn at baseline, 10 min and 30 min after ROSC, and every hour thereafter for blood gas analysis (ABL 700, Radiometer, Copenhagen, Denmark) and the collection of serum samples. Blood count analysis was performed at baseline and 6 h after ROSC (Celltac-alpha VET MEK-6550 K, Nihon Koden, Rosbach, Germany). Alanine transaminase (ALT), aspartate transaminase (AST), albumin, creatinine, creatine kinase (CK), and creatine kinase-MB (CK-MB) levels were measured in sera (Laboratory of Hematology at the Institute of Laboratory Animal Science and Experimental Surgery, RWTH Aachen University, Faculty of Medicine, Aachen, Germany). S100B and myoglobin levels were analysed using commercially available ELISA kits (porcine S100B (MBS2501779) and porcine myoglobin (MBS9425685), Biotrend, Cologne, Germany).

### Statistical analysis

GraphPad PRISM 9 (GraphPad Software, San Diego, CA, USA) was used for statistical analysis and graphical presentation of the data. The data are presented as the means ± standard deviations (SDs) unless otherwise stated. The normality of the data distribution was assessed using diagnostic plots and the Kolmogorov‒Smirnov test. Two-way analysis of variance (ANOVA) or a mixed model with Greenhouse–Geisser correction followed by Sidak’s test for multiple comparisons was applied for continuous variables. A t test was used for comparisons of two values. Categorical variables were analysed using the chi^2^ test. The null hypothesis was rejected at *p* < 0.05 (*p < 0.05, **p < 0.01, ***p < 0.001).

## Results

No relevant differences in weight, haemodynamic parameters, blood gas results or serum indicator levels were observed at baseline between the animals in the ICPR group and those in the ECPR group. Echocardiography confirmed that ventricular function was comparable between the experimental groups.

Preparation of the animals for the experiment was performed without any complications, and CA was successfully induced electrically in all the animals. The cannulation time for the arterial and venous ECMO cannulas (469 ± 129 s) was significantly longer than that for the single arterial pLVAD introducer (153 ± 64 s) (*p* < 0.001). Since ECMO cannulation and pLVAD introducer placement were performed before the induction of CA while the heart was beating, the duration did not impact resuscitation outcomes. The implantation time for the Impella ECP device via the previously placed femoral introducer sheath was 58 ± 31 s. Therefore, ECMO was initiated 60 s after the start of CPR to achieve a comparable no-flow time in both groups.

### MCS during resuscitation

**Fig. 2:**
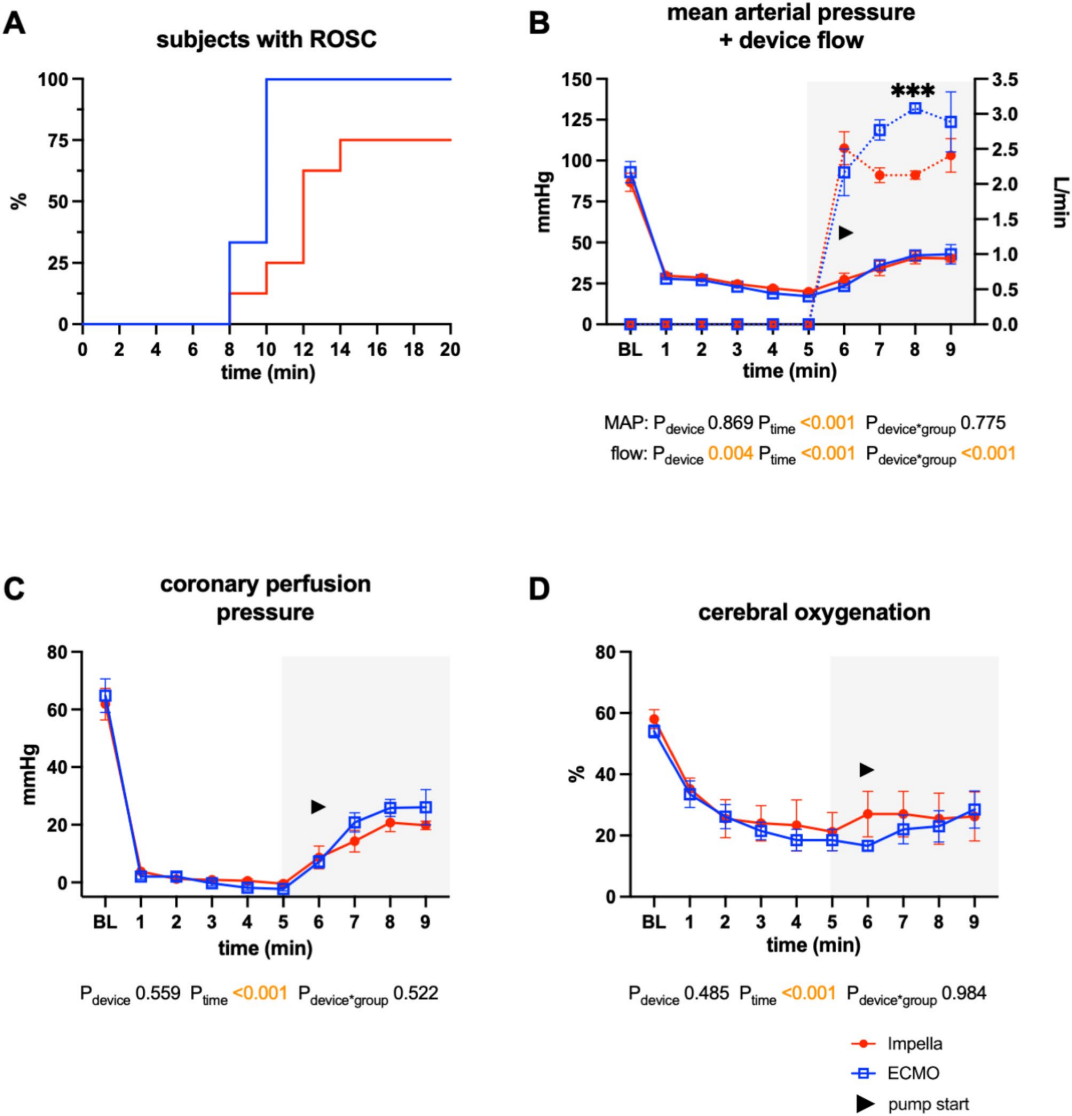
Resuscitation data of 8 swine treated with Impella ECP and 6 swine treated with veno-arterial ECMO. (**A**) Percentage of subjects with ROSC over time. (**B**) Mean arterial blood pressure and Impella/ECMO device flow. (**C**) Calculated coronary perfusion pressure. (**D**) Cerebral oxygenation. The data are presented as the means ± standard errors of the means. *BL* baseline; *ECMO* extracorporeal membrane oxygenation; *MAP* mean arterial pressure; *ROSC* return of spontaneous circulation.

Within the first 3 mins of CA, the mean arterial pressure (MAP), central venous pressure (CVP) and pulmonary arterial pressure (PAP) equalized (Fig. 2), and the calculated CPP decreased to < 1 mmHg. After the initiation of MCS and vasopressor administration, the MAP and calculated CPP increased similarly in both groups (*p* > 0.99 until 4 min after the intervention started). Although the ECMO flow was greater than the pLVAD flow at the beginning of MCS (Fig. 2B), neither the MAP nor the calculated CPP was significantly greater in the ECMO group (Fig. 2B,C). No difference in cerebral oxygenation measured by NIRS was observed during resuscitation between the two groups (Fig. 2D).

75% of the animals supported by Impella ECP achieved ROSC (6/8), whereas all animals resuscitated with ECMO exhibited ROSC (6/6) (survival, ICPR vs. ECPR, *p* = 0.186) (Fig. 2A). In one unsuccessfully resuscitated ICPR animal, Impella ECP damage during insertion led to inadequate device flow; in the other unsuccessfully resuscitated ICPR animal, ROSC was not established within 10 min of resuscitation. ECPR required less vasopressors during resuscitation (ICPR vs. ECPR: 1.8 ± 0.4 vs. 1.0 ± 0 mg norepinephrine, *p* = 0.015) and demonstrated a trend towards the need for fewer defibrillations (3.5 ± 2.0 vs. 2.2 ± 0.4, *p* = 0.136).

### MCS after ROSC

**Fig. 3:**
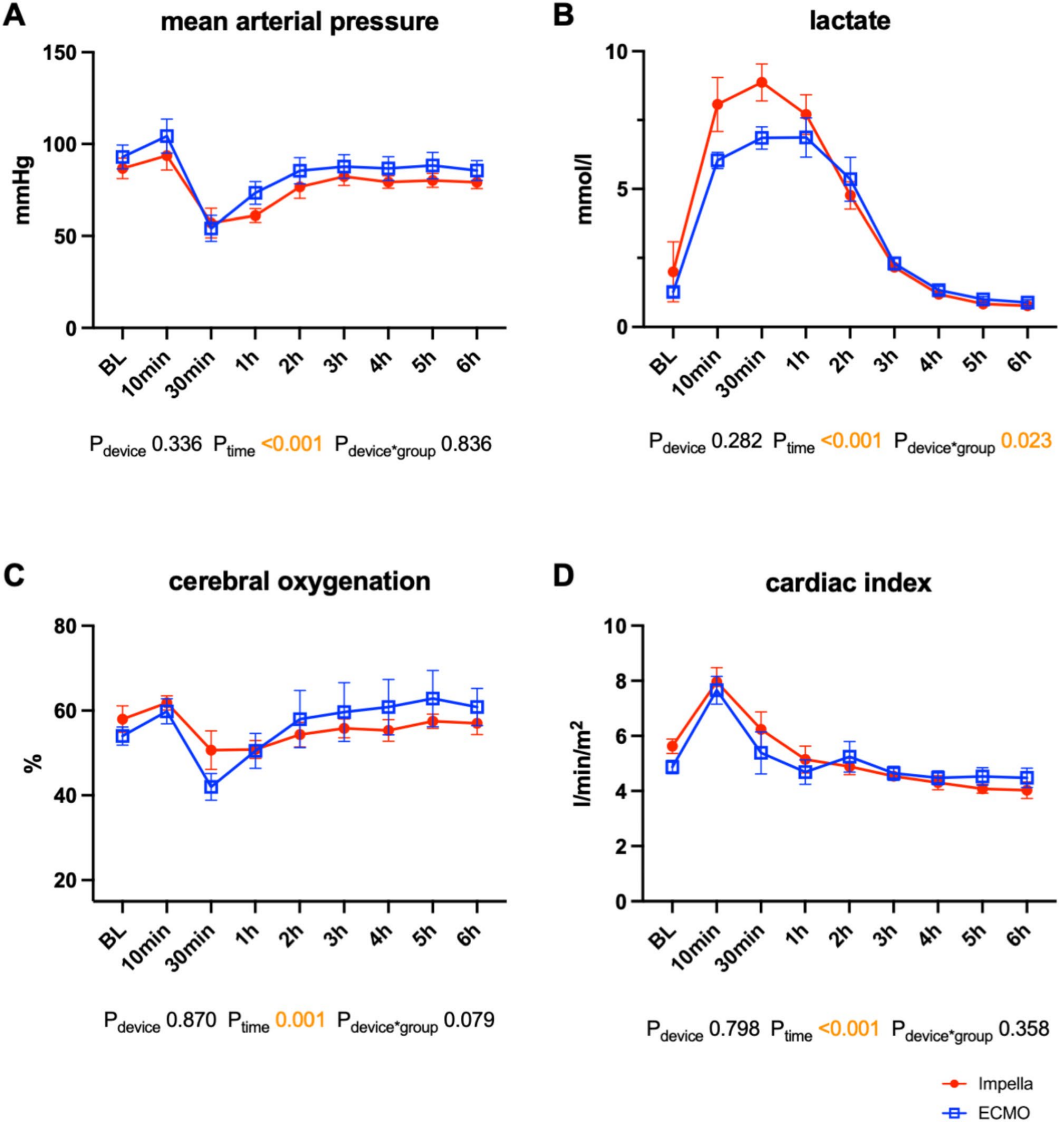
Post-resuscitation data of swine supported either by the Impella ECP (n = 6) or veno-arterial ECMO (n = 6). (**A**) Mean arterial blood pressure. (**B**) Lactate. (**C**) Cerebral tissue oxygenation. (**D**) Cardiac index measured by pulmonary thermodilution (CI in the ECMO group containing the ECMO device flow). The data are presented as the means ± standard errors of the means. *BL* baseline, *ECMO* extracorporeal membrane oxygenation.

**Fig. 4:**
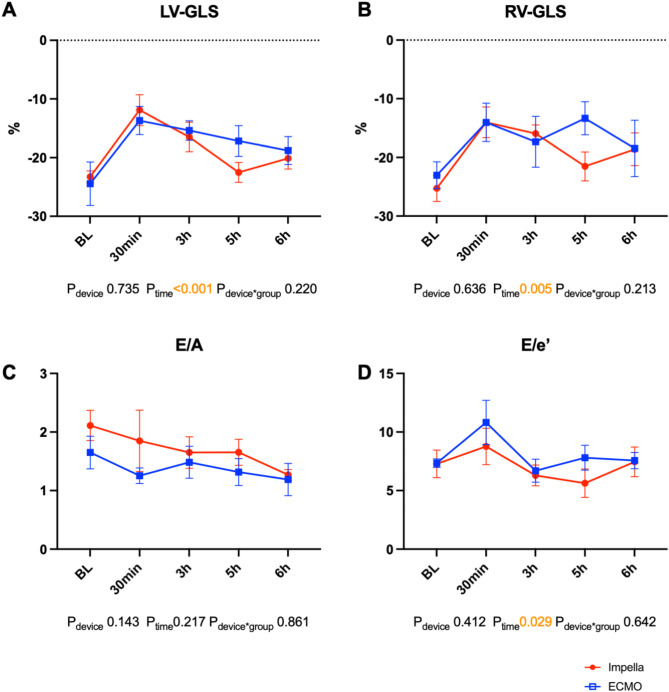
Echocardiographic parameters of 8 swine treated with Impella ECP and 6 swine treated with veno-arterial ECMO. (**A**) Left-ventricular global longitudinal strain. (**B**) Right-ventricular global longitudinal strain. (**C**) E/A ratio. (**D**) E/e’ ratio. The data are presented as the means ± standard errors of the means. *BL* baseline, *ECMO* extracorporeal membrane oxygenation, *GLS* global longitudinal strain, *LV* left ventricle, *RV* right ventricle.

After ROSC, the animals in both groups stabilized without the use of vasopressors. Temporal tachycardia, mild arterial hypotension and lactatemia were observed in the animals in both treatment groups (Fig. 3A,B; Table 1). Cerebral oxygenation mildly decreased 30 min post-ROSC, which was associated with mild hypotension (Fig. 3A,C). The Swan Ganz catheter-derived cardiac index (CI) of the pulmonary circulation was greater in the ICPR group (ICPR vs. ECPR PR 10:5.5 ± 0.8 L/min vs. 4.9 ± 0.6 L/min, *p* = 0.03; PR 300:4.1 ± 0.4 L/min vs. 2.9 ± 0.4 L/min, *p* = 0.003). The flow generated by VA-ECMO was added to the pulmonary CI to estimate the systemic CI, and no differences were observed between the two treatment groups (Fig. 3D). Haemodynamics and blood gas values were stable in both groups after termination of MCS 5 h post ROSC. Echocardiography revealed a similar restriction of LV and RV systolic function after ROSC in both groups (Fig. 4A,B, Table 2; LV-global longitudinal strain [GLS] 3 h post-ROSC ICPR: -16.5 ± 5.6% vs. ECPR: − 13.7 ± 5.9%, *p* = 0.99; RV-GLS 3 h post-ROSC ICPR: − 15.9 ± 3.3% vs. ECPR: − 17.3 ± 10.6%, *p* = 0.99), which recovered during the observation period. No relevant differences were observed in the parameters of diastolic dysfunction (Fig. 4C,D). Together with native cardiac output, both treatments generated sufficient systemic perfusion, resulting in adequate lactate clearance and haemodynamic stabilization (Table 1). Lactate levels returned to baseline within 4 h post-ROSC in both groups (*p* > 0.99). ALT, creatinine, CK and CK-MB levels were not different between the ICPR and ECPR groups (Table 3). However, AST levels increased post ROSC in the ICPR group (10 min post-ROSC ICPR vs. ECPR: *p* = 0.028; Table 3). S100B and myoglobin levels did not differ substantially between ICPR and ECPR groups (S100B *p* = 0.27; myoglobin *p* = 0.19).

**Table 1:** Haemodynamic and blood gas data.

	BL Impella = 8 ECMO = 6	PR 10 Impella = 6 ECMO = 6	PR 30 Impella = 6 ECMO = 6	PR 60 Impella = 6 ECMO = 6	PR 120 Impella = 6 ECMO = 6	PR 300 Impella = 6 ECMO = 6	PR 360 Impella = 6 ECMO = 6
HR [bpm]							
Impella	73 ± 15	144 ± 33	134 ± 43	105 ± 5	86 ± 10	67 ± 11	70 ± 17
ECMO	59 ± 5	143 ± 24	112 ± 11	91 ± 14	81 ± 15	64 ± 14	71 ± 16
MAP [mmHg]							
Impella	86 ± 12	94 ± 19	57 ± 20	61 ± 9	77 ± 15	80 ± 9	79 ± 8
ECMO	93 ± 16	104 ± 23	54 ± 17	73 ± 15	85 ± 17	88 ± 18	86 ± 13
MPAP [mmHg]							
Impella	21 ± 1	23 ± 5	21 ± 4	22 ± 3	21 ± 3	23 ± 2	22 ± 2
ECMO	23 ± 2	20 ± 4	21 ± 6	21 ± 3	21 ± 3	21 ± 4	22 ± 2
PCWP [mmHg]							
Impella	14 ± 3	15 ± 3	15 ± 2	15 ± 2	15 ± 2	14 ± 2	13 ± 2
ECMO	15 ± 2	14 ± 3	15 ± 2	16 ± 3	14 ± 2	13 ± 2	14 ± 2
CVP [mmHg]							
Impella	10 ± 2	11 ± 3	12 ± 4	12 ± 2	11 ± 2	12 ± 2	11 ± 2
ECMO	11 ± 1	10 ± 4	11 ± 2	11 ± 2	10 ± 2	9 ± 2	10 ± 1
S_c_O_2_ [%]							
Impella	57 ± 7	62 ± 4	51 ± 11	51 ± 5	54 ± 7	58 ± 4	57 ± 7
ECMO	52 ± 5	60 ± 7	42 ± 8	51 ± 10	58 ± 17	63 ± 16	61 ± 11
CI [L/m^2^]							
Impella	5.5 ± 0.8	8 ± 1.3	6.2 ± 1.6	5.1 ± 1.2	4.9 ± 0.7	4.1 ± 0.4	4.0 ± 0.7
ECMO_†_	4.9 ± 0.6	7.7 ± 1.2	5.4 ± 1.9	4.7 ± 1.1	5.2 ± 1.4	4.5 ± 0.8	4.5 ± 0.9
ECMO	4.9 ± 0.6	5.4 ± 1.1	3.4 ± 1.9	2.9 ± 0.7	3.6 ± 1.2	2.9 ± 0.4	4.5 ± 0.9
Device flow [L/min]							
Impella		2.9 ± 0.5	3.3 ± 0.9	3.4 ± 0.4***	3.0 ± 0.8	3.0 ± 0.6*	
ECMO		2.7 ± 0.4	2.3 ± 0.5	2.0 ± 0.3***	1.9 ± 0.4	2.0 ± 0.3*	
S_v_O_2_ [%]							
Impella	62 ± 8	82 ± 4	53 ± 16	51 ± 11	53 ± 9	52 ± 6	54 ± 9
ECMO	60 ± 6	85 ± 10	48 ± 10	59 ± 9	55 ± 5	56 ± 9	50 ± 6
pH							
Impella	7.47 ± 0.03	7.30 ± 0.05	7.34 ± 0.2	7.38 ± 0.05	7.45 ± 0.04	7.49 ± 0.00	7.49 ± 0.01
ECMO	7.50 ± 0.02	7.34 ± 0.02	7.30 ± 0.05	7.37 ± 0.10	7.44 ± 0.07	7.46 ± 0.03	7.47 ± 0.02
PaO_2_ [mmHg]							
Impella	144 ± 20	472 ± 94	173 ± 38	162 ± 19	161 ± 10	166 ± 12	171 ± 14
ECMO	149 ± 20	456 ± 35	121 ± 63	162 ± 62	134 ± 47	119 ± 31	144 ± 26
PaCO_2_ [mmHg]							
Impella	38 ± 2	42 ± 3	40 ± 4	38 ± 4	37 ± 3	37 ± 1	38 ± 1
ECMO	39 ± 2	45 ± 4	49 ± 5	42 ± 9	38 ± 5	42 ± 3	40 ± 2
Lactate (mmol/L)							
Impella	1.7 ± 2.3	8.1 ± 2.4	8.9 ± 1.7	7.7 ± 1.8	4.8 ± 1.2	0.8 ± 0.1	0.8 ± 0.1
ECMO	1.3 ± 0.4	6.0 ± 0.7	6.9 ± 1.0	6.9 ± 1.8	5.4 ± 2.0	1.0 ± 0.3	0.9 ± 0.2

Haemodynamic and blood gas values of 14 swine that were resuscitated with either the Impella ECP or veno-arterial ECMO at baseline (BL) and at 10 (PR 10), 30 (PR 30), 60 (PR 60), 120 (PR 120), 300 (PR 300) and 360 (PR 360) minutes following the return of spontaneous circulation.

*CVP* central venous pressure; *CI* cardiac index; *ECMO* extracorporeal membrane oxygenation; *ECMO*_*†*_ extracorporeal membrane oxygenation pulmonary cardiac index + ECMO flow (p values for Impella vs. ECMO_†_); *HR* heart rate; *MAP* mean arterial pressure; *MPAP* mean pulmonary artery pressure; *PaCO*_*2*_ arterial CO_2_ partial pressure; *PaO*_*2*_ arterial O_2_ partial pressure; *PLT* platelets; *PCWP* pulmonary capillary wedge pressure; *S*_*c*_*O*_*2*_ cerebral tissue oxygen saturation; *S*_*v*_*O*_*2*_ mixed venous oxygenation. The data are presented as the means ± standard deviations.

*p* > 0.05 unless indicated otherwise, *p < 0.05, ***p < 0.001.

**Table 2:** Echocardiographic left and right ventricular function parameters.

	BL Impella = 7 ECMO = 6	PR 30 Impella = 5 ECMO = 6	PR 180 Impella = 5 ECMO = 6	PR 300 Impella = 5 ECMO = 6	PR 360 Impella = 5 ECMO = 6
LV-EF [%]					
Impella	59 ± 7	45 ± 9	57 ± 6	67 ± 10	59 ± 10
ECMO	69 ± 7	53 ± 13	56 ± 15	58 ± 11	60 ± 11
LV-GLS [%]					
Impella	− 24.2 ± 3.3	− 11.9 ± 5.8	− 16.5 ± 5.6	− 22.5 ± 3.8	− 20.1 ± 4.1
ECMO	− 24.1 ± 9.0	− 13.7 ± 5.9	− 15.4 ± 4.1	− 17.2 ± 6.4	− 18.8 ± 5.8
RV-GLS [%]					
Impella	− 26.2 ± 4.4	− 14.0 ± 5.9	− 15.9 ± 3.3	− 21.5 ± 5.5	− 18.6 ± 6.2
ECMO	− 22.0 ± 5.6	− 14.0 ± 8.0	− 17.3 ± 10.6	− 13.3 ± 6.9	− 18.5 ± 11.8
RVD_1_ [mm]					
Impella	28 ± 5	30 ± 7	31 ± 6	30 ± 6	29 ± 4
ECMO	29 ± 5	25 ± 3	28 ± 4	30 ± 2	28 ± 4
TASV [cm/s]					
Impella	11 ± 2	7 ± 3	7 ± 1	8 ± 2	9 ± 2
ECMO	11 ± 2	8 ± 4	7 ± 3	7 ± 2	8 ± 1
MV DT [ms]					
Impella	253 ± 151	130 ± 80	143 ± 26	191 ± 77	213 ± 65
ECMO	189 ± 67	96 ± 15	173 ± 34	159 ± 70	152 ± 37
E/A					
Impella	1.8 ± 0.8	1.8 ± 1.2	1.7 ± 0.5	1.7 ± 0.5	1.3 ± 0.2
ECMO	1.7 ± 0.7	1.3 ± 0.3	1.5 ± 0.7	1.3 ± 0.6	1.2 ± 0.7
E/e’					
Impella	7.1 ± 2.3	8.8 ± 3.5	6.3 ± 1.8	5.6 ± 2.7	7.5 ± 2.8
ECMO	7.3 ± 0.9	10.8 ± 4.6	6.7 ± 2.4	7.8 ± 2.6	7.6 ± 1.7

Echocardiographic data of 13 swine that were resuscitated with either the Impella ECP or veno-arterial ECMO at baseline (BL) and at 30 (PR 30), 180 (PR 180), 300 (PR 300) and 360 (PR 360) minutes following the return of spontaneous circulation. The data are presented as the means ± standard deviations.

*E/A* early mitral inflow/mitral inflow at atrial contraction, *E/e’* early mitral inflow/mitral annular early diastolic velocity, *LV-EF* left ventricular ejection fraction, *LV-GLS* left ventricular global longitudinal strain, *MV DT* mitral valve deceleration time, *RVD*_*1*_ right ventricular basal diameter, *RV-GLS* right ventricular global longitudinal strain, *TASV* tricuspid annular systolic velocity.

*p* > 0.05 for all comparisons.

**Table 3:** Serum parameters.

	BL Impella = 8 ECMO = 6	PR 10 Impella = 6 ECMO = 6	PR 30 Impella = 6 ECMO = 6	PR 60 Impella = 6 ECMO = 6	PR 360 Impella = 6 ECMO = 6
AST [U/L]					
Impella	28 ± 8	85 ± 24*	73 ± 10	77 ± 10	52 ± 32
ECMO	22 ± 9	40 ± 16*	50 ± 23	51 ± 23	38 ± 8
ALT [U/L]					
Impella	51 ± 15	97 ± 54	84 ± 41	74 ± 36	48 ± 10
ECMO	54 ± 7	54 ± 4	54 ± 4	55 ± 5	54 ± 9
Crea [µmol/L]					
Impella	145 ± 21	176 ± 27	185 ± 32	194 ± 39	171 ± 25
ECMO	123 ± 15	141 ± 13	143 ± 19	145 ± 25	159 ± 25
Albumin [g/L]					
Impella	31 ± 2	29 ± 2*	28 ± 2***	28 ± 2**	27 ± 2*
ECMO	25 ± 5	22 ± 4*	21 ± 2***	20 ± 3**	20 ± 5*
CK [U/L]					
Impella	574 ± 216	1337 ± 670	1249 ± 510	1497 ± 673	5241 ± 3971
ECMO	950 ± 671	1152 ± 474	1164 ± 580	1274 ± 829	3517 ± 1615
CK-MB [U/L]					
Impella	402 ± 348	1143 ± 753	1005 ± 539	1182 ± 433	3811 ± 2370
ECMO	745 ± 469	1186 ± 787	938 ± 422	985 ± 504	2402 ± 999
S100B [ng/mL]					
Impella	5.9 ± 2	6 ± 1.8		14.8 ± 8.8	6.2 ± 2.3
ECMO	5.8 ± 1.2	10.2 ± 3.7		13.9 ± 3.8	6.4 ± 2.4
Myo [ng/mL]					
Impella	332 ± 76	323 ± 55		272 ± 57	263 ± 77
ECMO	379 ± 63	339 ± 127		361 ± 95	369 ± 106
PLT [nl^−1^]					
Impella	280 ± 65				334 ± 163
ECMO	204 ± 37				244 ± 88

Blood parameters of 14 swine that were resuscitated with either the Impella ECP or veno-arterial ECMO at baseline (BL), 10 (PR 10), 30 (PR 30), 60 (PR 60) and 360 (PR 360) minutes following the return of spontaneous circulation.

*ALT* alanine transaminase, *AST* aspartate transaminase, *Crea* creatinine, *CK* creatine kinase, *CK-MB* creatine kinase-MB, *PLT* platelet count, *S100B* S100 calcium binding protein B. Data are presented as the means ± standard deviations.

*p* > 0.05 if not indicated otherwise, *p < 0.05, **p < 0.01, ***p < 0.001.

All successfully resuscitated animals (12/12) survived until the end of the experiment. No ischaemic complication or bleeding caused by vascular access was observed in the ICPR group. In the ECPR group, limb ischaemia was detected in 1 of 6 animals.

## Discussion

This exploratory animal study aimed to compare the effects of MCS by pLVAD and peripheral VA-ECMO during CA and in the post-resuscitation period. Although VA-ECMO is clinically used for resuscitation, there is clearly a need for additional research and refinement of this highly invasive technology. In this study, both therapeutic options successfully facilitated ROSC after defibrillation and provided adequate haemodynamic stabilization after ROSC without the use of catecholamines, ultimately resulting in successful weaning from MCS in all resuscitated subjects.

The effectiveness of MCS during CA has been demonstrated for both transvalvular microaxial pumps^5,8,9,20,21^ and peripheral VA-ECMO^22,23^. While ECPR has gained acceptance in clinical practice^24^, the utilization of pLVADs is rare and is based on the individual decisions of attending physicians. From a mechanistic perspective, the two mechanical circulatory devices differ in the direction of the generated blood flow, and there is a possibility of additional oxygenation, decarboxylation and temperature control when ECMO is employed. Both devices induce a non-pulsatile flow that is influenced by the remaining cardiac activity and the degree of mechanical support. However, ICPR involves blood withdrawal from the LV and anterograde delivery to the ascending aorta after passive passage through the pulmonary circulation. The passive pulmonary blood circulation is known to limit the maximum achievable blood flow of the Impella device. In a previous study by our group, the maximum Impella flow during resuscitation could be increased by the application of inhaled nitric oxide, a pulmonary vasodilator^6^. Lotun et al*.* and Gottula et al. reported greater achievable pLVAD flows and greater CPPs when pLVADs were combined with manual chest compressions than when pLVADs were used alone^25,26^. This finding is consistent with observations from this study, in which ECMO was able to generate a greater blood flow during resuscitation, although the Impella ECP device utilized can theoretically generate equally high flow rates with optimal LV filling. Packer et al*.* compared an LV Impella device with a biventricular Impella device during CA. They reported a greater pLVAD flow during biventricular support but a lower LV myocardial perfusion pressure and lower ROSC rates than those of the LV Impella device alone^27^. Although blood withdrawal from the LV by the Impella device has been shown to increase CPP^28^, which should facilitate the likelihood of successful defibrillation, our study did not show a favourable outcome regarding the probability of successful defibrillation in the ICPR group.

ECMO support requires two vascular cannulas, necessitating a longer cannulation time than that required for the single vascular access point for ICPR. In our study, placement of the pLVAD took approximately 2.5 min, whereas the placement of both ECMO cannulas took almost 8 min (*p* < 0.001). The effect of the longer ECMO cannulation time on the resuscitation outcome was excluded in this study since cannulation was performed before CA was induced. However, in a clinical scenario, a longer cannulation time would be associated with a longer low-flow state, most likely translating into an adverse resuscitation outcome. In addition, outside of a controlled experimental setting, a high rate of vascular complications can likely be expected during cannulation in an emergency. Reliable data on vascular complications such as bleeding, dissection or limb ischaemia for implantation of both devices during CA are currently lacking. When used during cardiogenic shock, vascular complications occur in 39.5% of patients with ECMO and 16.7% of patients with the Impella device (*p* = 0.001)^13,29^. In our study, no bleeding occurred during cannulation, but limb ischaemia was observed in one of the ECPR animals.

After ROSC, the haemodynamic characteristics of the devices change: systemic perfusion is no longer solely dependent on the output of the mechanical devices but rather enhanced by them. In cases where CA is triggered by a myocardial infarction, LV unloading before reperfusion may reduce the extent of the myocardial scar^30–33^, a benefit that is not achievable with ECMO^34^. In comparison, ECMO can increase LV afterload, leading to increased myocardial work and oxygen consumption^35,36^. In contrast, the Impella device reduces LV wall tension, thereby diminishing microvascular resistance and enhancing myocardial perfusion^28,37^. Consequently, quicker recovery of LV function could be expected in Impella-treated animals. However, we did not observe any significant difference in LV echocardiographic function or pulmonary capillary wedge pressure (PCWP) between the two treatment groups post-ROSC, indicating that there were only minor differences in myocardial dysfunction^36,38^. This lack of disparity may be attributed to the relatively short absolute ischaemia time of less than 10 min and the utilization of young and healthy animals. This result would also explain why patients present more frequently with cardiogenic shock after ECPR, a situation that can be resolved by using an LV Impella in addition to ECMO^39–41^.

Several limitations should be considered when interpreting the findings of this trial. First, out-of-hospital CA tends to occur in elderly individuals with pre-existing conditions such as coronary artery disease or heart failure. In contrast, the animals in this study were young and free from preexisting conditions. However, replicating the complexity of preexisting conditions in an animal model is inherently challenging. Second, we chose to deviate from a randomized approach for the study design because of the need for comparable ischaemia times for both treatment groups. We contend that a randomized experimental design with unequal ischaemia times in both groups would have led to a significant bias regarding the comparability of the haemodynamic effects. Longer no-flow times lead to a reduction on the CPP and the ROSC rates. Both the arterial sheath for the pLVAD and the ECMO cannulas were implanted prior to the induction of CA. In this respect, our approach differs from the clinical setting, limiting its direct transferability. Furthermore, in a clinical setting chest compressions would have been performed before MCS was inducted, that were not part of our study protocol. Finally, our study does not offer insights into neurological outcomes after resuscitation. Owing to the haemodynamic focus of the study, the experimental protocol involved only a short period of untreated global ischaemia of approximately 5 min. Cerebral sequelae were expected to be relatively minor in both groups, leading to challenges in detecting clinically relevant differences. Therefore, we refrained from conducting a final neurological examination of the animals without anaesthesia.

## Conclusions

This experimental study compared the effects of LV Impella- and VA-ECMO-based MCS during cardiopulmonary resuscitation and subsequent ROSC. Both the Impella device and VA-ECMO generated adequate perfusion pressure during resuscitation, facilitating ROSC. Post-ROSC, both devices provided sufficient circulatory support, and all resuscitated animals survived until the end of the study. No significant hemodynamic differences were detected between the treatments. More research is needed to determine whether there are indications where patients may benefit from one of these devices rather than the other.

## Data Availability

The authors declare that they have full control of all primary data, and they agree to allow the journal to review their data if requested. The dataset is available from the corresponding author on reasonable request.

## Abbreviations

ALT: Alanine transaminase
AST: Aspartate transaminase
BSA: Body surface area
CA: Cardiac arrest
CI: Cardiac index
CK: Creatine kinase
CPP: Coronary perfusion pressure
CVP: Central venous pressure
ECG: Electrocardiogram
ECPR: Extracorporeal cardiopulmonary resuscitation
GLS: Global longitudinal strain
ICPR: Intravascular cardiopulmonary resuscitation
LV: Left ventricle
MAP: Mean arterial pressure
MCS: Mechanical circulatory support
NIRS: Near-infrared spectroscopy
PAP: Pulmonary arterial pressure
pLVAD: Percutaneous left ventricular assist device
ROSC: Return of spontaneous circulation
RV: Right ventricle
SD: Standard deviation
VA-ECMO: Veno-arterial extracorporeal membrane oxygenation
VF: Ventricular fibrillation
